# Celecoxib for Mood Disorders: A Systematic Review and Meta-Analysis of Randomized Controlled Trials

**DOI:** 10.3390/jcm12103497

**Published:** 2023-05-16

**Authors:** Adam Gędek, Zofia Szular, Anna Z. Antosik, Paweł Mierzejewski, Monika Dominiak

**Affiliations:** 1 Department of Pharmacology, Institute of Psychiatry and Neurology, Sobieskiego 9, 02-957 Warsaw, Poland; adamgedek@gmail.com (A.G.);; 2 Praski Hospital, Aleja Solidarności 67, 03-401 Warsaw, Poland; 3 Faculty of Medicine, Medical University of Warsaw, Żwirki i Wigury 61, 02-091 Warsaw, Poland; 4 Department of Psychiatry, Faculty of Medicine, Collegium Medicum, Cardinal Wyszynski University in Warsaw, Woycickiego 1/3, 01-938 Warsaw, Poland

**Keywords:** celecoxib, cyclooxygenase 2 inhibitor, COX-2, mood disorders, bipolar disorder, major depression, depressive episode, mania, animal model of mania, animal model of depression

## Abstract

The effects of celecoxib on a broad spectrum of mood disorders and on inflammatory parameters have not yet been comprehensively evaluated. The aim of this study was to systematically summarize the available knowledge on this topic. Data from both preclinical and clinical studies were analyzed, considering the efficacy and safety of celecoxib in the treatment of mood disorders, as well as the correlation of inflammatory parameters with the effect of celecoxib treatment. Forty-four studies were included. We found evidence supporting the antidepressant efficacy of celecoxib in a dose of 400 mg/day used for 6 weeks as an add-on treatment in major depression (SMD = −1.12 [95%Cl: −1.71,−0.52], *p* = 0.0002) and mania (SMD = −0.82 [95% CI:−1.62,−0.01], *p* = 0.05). The antidepressant efficacy of celecoxib in the above dosage used as sole treatment was also confirmed in depressed patients with somatic comorbidity (SMD = −1.35 [95% CI:−1.95,−0.75], *p* < 0.0001). We found no conclusive evidence for the effectiveness of celecoxib in bipolar depression. Celecoxib at a dose of 400 mg/d used for up to 12 weeks appeared to be a safe treatment in patients with mood disorders. Although an association between celecoxib response and inflammatory parameters has been found in preclinical studies, this has not been confirmed in clinical trials. Further studies are needed to evaluate the efficacy of celecoxib in bipolar depression, as well as long-term studies evaluating the safety and efficacy of celecoxib in recurrent mood disorders, studies involving treatment-resistant populations, and assessing the association of celecoxib treatment with inflammatory markers.

## 1. Introduction

Major depressive disorder (MDD) and bipolar disorder (BD) are mood disorders that impair the quality of life and shorten life expectancy [1]. Studies report that MDD and BD affect, respectively, 246 and over 39 million people globally [2,3]. 

Due to a high rate of treatment resistance, novel therapies are being considered [4,5,6,7,8,9,10]. Nevertheless, insufficient knowledge of the etiology is a limiting factor in obtaining a satisfactory treatment. Numerous correlations and mechanisms have been studied to understand the pathophysiology of mood disorders [11,12,13]. Of particular note is the hypothesis that includes the role of inflammatory background in mood disorders. It comes from studies of comorbidity between mood disorders and chronic inflammatory disorders, neuroimaging, levels of pro-inflammatory and anti-inflammatory biomarkers, as well as post-mortem brain studies [14,15,16,17,18,19,20,21,22,23,24,25].

Regarding inflammatory biomarkers, patients with MDD had significantly higher mean levels of IL-1β, IL-3, IL-6, IL-10, IL-12, IL-18, the soluble IL-2 receptor (sIL-2R), and tumor necrosis factor α (TNFα) compared to healthy controls [26,27,28]. Patients with BD also exhibited increased concentrations of CRP, IL-4, IL-10, and decreased concentrations of brain-derived neurotrophic factor (BDNF) [27,29,30].

Among anti-inflammatory agents, cyclooxygenase-2 inhibitors are a potential treatment for mood disorders [31,32,33,34]. A representative of this class of drugs is celecoxib, which belongs to the non-steroidal anti-inflammatory drugs (NSAIDs) [35]. NSAIDs inhibit the enzyme cyclooxygenase (COX), which converts arachidonic acid into prostanoids via two distinct isozymes, COX-1 and COX-2. COX-1 helps maintain gastrointestinal mucosa lining. COX-2 plays a role in inflammation; it synthesizes prostaglandin E2 (PGE2), which acts as a stimulator of indoleamine-2,3-dioxygenase (IDO) and mediates inflammatory response [35,36]. Thereby, selective cyclooxygenase-2 inhibitors provide anti-inflammatory effects without causing stomach irritation. Moreover, according to research, celecoxib has a better cardiovascular safety profile in comparison with ibuprofen or naproxen [37,38].

In recent years, several reviews and meta-analyses have been published on the use of celecoxib for mood disorders. These mainly concerned depression [34,39,40], with one study focused on mania [41]. The Kittur et al. (2022) study, a scoping review, analyzed celecoxib in patients with MDD and bipolar depression. Since conflicting results were found, the authors suggested the need to stratify patients according to the inflammation status and clinical presentation [39]. On the other hand, a meta-analysis by Wang et al. (2022) showed that celecoxib was effective in treating major depression in the course of both bipolar and unipolar disorder [40]. As in the Kittur et al. (2022) study, it was found that the type of depression was a possible source of variation in efficacy results. Since in this study the inflammatory markers were not evaluated, they recommended that future meta-analyses should take into account depression type along with inflammation markers. Finally, in a recent systematic review of meta-analyses of anti-inflammatory agents (including celecoxib) in MDD, it was stated that no clear-cut recommendations can be made due to the heterogeneity of patient populations. The authors emphasized the need to identify anti-inflammatory biomarkers in given populations of patients with depression for more tailored therapy [34]. As regards the use of celecoxib for affective conditions other than depression, we identified only one systematic review and meta-analysis highlighting its potential efficacy in mania [41].

Given the conclusions of the reviews and meta-analyses cited above, as well as the postulated inflammatory background in a wide range of mood disorders, a combined analysis and comparison is relevant. We decided to bring together in a single paper the broadest possible spectrum of mood disorders, including different types of depression and different affective episodes with a simultaneous attempt to identify inflammatory markers in given types of depression and in given affective episodes. Thus, this review analyzed the effect of celecoxib’s efficacy and safety according to diagnosis, including major depression, bipolar depression and depressive symptoms in somatic disorders, and including all affective episodes (both mania and depression). We also aimed to investigate the association of celecoxib treatment with inflammatory markers in a given type of depression and affective state. In addition, we also pooled preclinical studies in models of depression and mania with clinical reports to make suggestions for future research. To the best of our knowledge, the effects of celecoxib on major depression, bipolar depression and mania, depressive symptoms in somatic disorders, and inflammatory parameters in preclinical and clinical studies have not yet been evaluated in such a comprehensive manner.

## 2. Materials and Methods

This systematic review was conducted according to the PRISMA statement (Preferred Reporting Items For Systematic Review and Meta-Analysis), based on previously prepared, unregistered protocol [42]. Two reviewers conducted each stage throughout the review process independently. Any disagreements between investigators were resolved via discussion and the opinion of a senior researcher to achieve a consensus.

### 2.1. Eligibility Criteria

Each relevant publication was evaluated using the PICO model (Table 1). Articles that met predefined criteria presented were included and categorized as preclinical and clinical (observational or interventional studies).

The included criteria were as follows: (1) preclinical, observational, or interventional study of any designs; (2) study on the effect of celecoxib on mood disorders or affective symptoms or for behavioral testing in an animal model of mood disorders; (3) participants over 18 years of age and under 65 years of age (applies to clinical studies); (4) published in English. The excluded criteria were as follows: (1) not conforming with PICO; (2) not an original article; (3) not in English; (4) full text was not available; (5) not published.

### 2.2. Data Acquisition and Search Strategy

We searched PubMed, Scopus, and Web of Science for studies published from inception to November 2022. We selected only databases that were accessible to reviewers through the institution. The search string used was (“bipolar disorder” or “bipolar depression” or “mania” or “hypomania” or “mixed episode” or “major depression” or “mood disorders”) and (“celecoxib” or “celebrex” or “4-(5-(4-methylphenyl)-3-(trifluoro methyl)-1H-pyrazol-1-yl) benzenesulfonamide”). Full search strategy for each database and registry are presented in Supplementary Material File S1. Follow-up citations were also scanned for relevant articles. After removing duplicates and reviewing titles and abstracts, the full text of all qualified studies were obtained to access the eligibility criteria.

### 2.3. Data Extraction

Data related to the effects of celecoxib on mood disorders were extracted independently using a tailored form. The form included: authors, year of publication, country, study design, sample and control size, duration, characteristics of the research and control group (sex, mean age, diagnosis, treatment), dose of celecoxib, and outcomes (impact on affective symptoms/behavioral tests, adverse effects, inflammatory markers).

### 2.4. Quality Assessment

Risk of bias of clinical trials was conducted in accordance with the Cochrane Collaboration guidelines [43] with RoB2 and ROBINS-I [44,45]. The Robvis tool was used for visualization [46]. A detailed description of the risk assessment is included in Supplementary Material File S1.

### 2.5. Synthesis and Analysis

Search results from Mendeley Desktop (version 1.19.8) have been transferred to Review Manager (RevMan5 version 5.4; Cochrane Collaboration). Continuous outcomes were pooled as standardized mean difference (SMD). Whenever the heterogeneity I^2^ test was below 75% the results were pooled. A fixed-effects model was used for the analysis. Studies with a risk of bias judged as “high” were excluded from the analysis. A subgroup analysis of treatment-resistant patients (TRD) was also planned. A detailed description of the synthesis and analysis is included in Supplementary Material File S1.

## 3. Results

### 3.1. Study Selections

**Figure 1:**
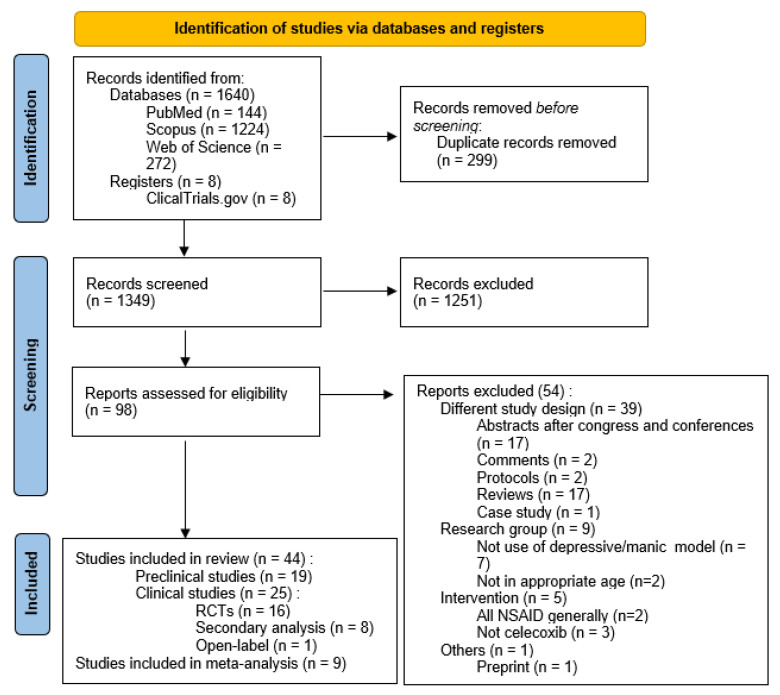
Flowchart showing an overview of the study selection process.

A total of 1640 papers were identified through the search strategy. After the removal of duplicates and exclusion based on titles or abstracts, 98 articles were screened in more detail for eligibility. Subsequently, another 54 were excluded, which resulted in the 44 publications used in this systematic review. This process is described in the PRISMA flowchart (Figure 1).

### 3.2. Description of Studies

The included studies were published between 2006 and 2021. Among identified studies, 19 were preclinical, 17 were interventional (16 randomized controlled trials, and 1 open-label study), and 8 were secondary analyses of RCTs. All preclinical studies involved rodents and used models of depression or mania. Clinical studies were published in the population of adults 18–65, two studies included patients up to 70 years old [47,48], and one up to 75 years old [49]. Study duration ranged from 6 to 12 weeks, with a mean of 6.5 weeks. The studies were conducted in the following countries: Iran (12), USA (8), Brazil (4), Germany (4), China (3), Canada (2), India (2), Italy (2), Netherlands (2), Australia (1), Denmark (1), Pakistan (1), Portugal (1), and Russia (1).

### 3.3. Quality Assesment

**Figure 2:**
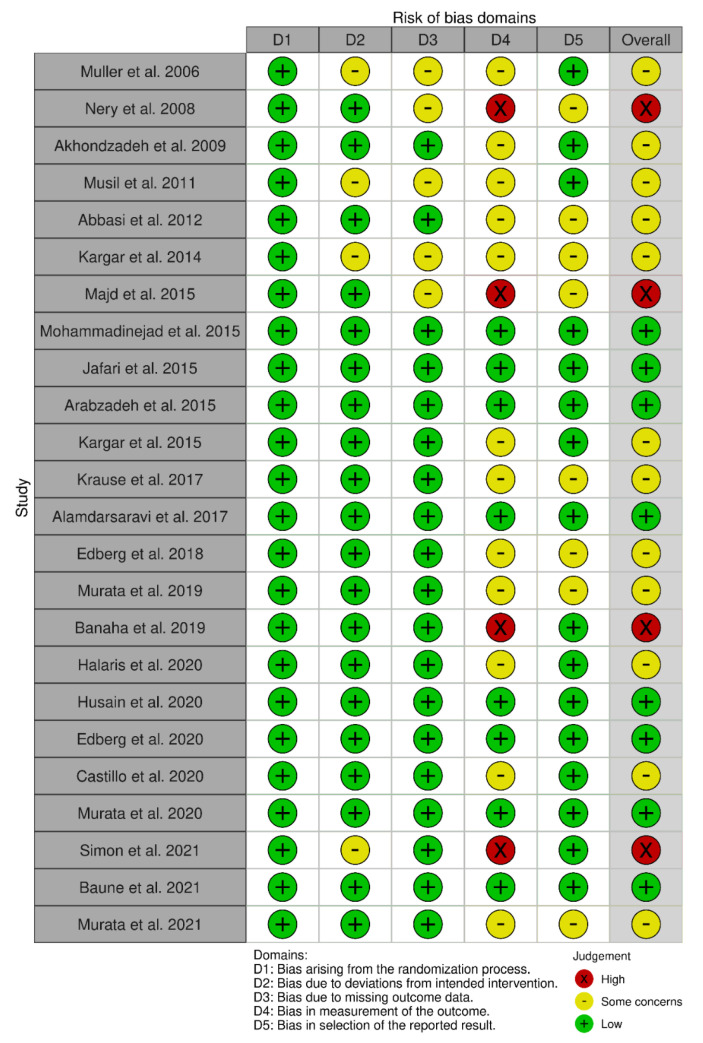
Risk of bias for interventional randomized studies with RoB2 tool [47,48,49,50,51,52,53,54,55,56,57,58,59,60,61,62,63,64,65,66,67,68,69,70].

**Figure 3:**
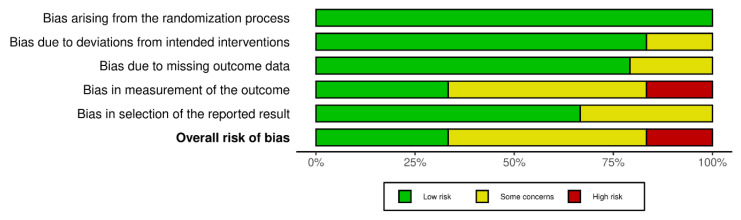
Risk of bias summary for interventional randomized studies with RoB2 tool.

**Figure 4:**
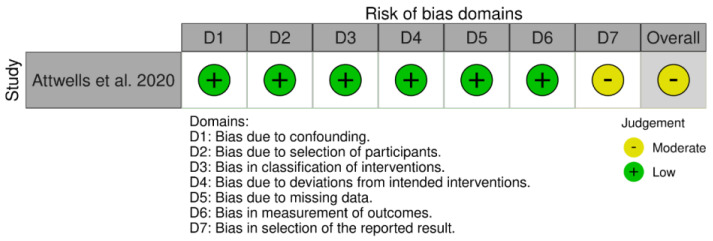
Risk of bias for interventional non-randomized study with ROBINS-I tool [71].

The interventional randomized controlled studies and secondary analysis of RCT with relevant outcomes were ranked according to the RoB2 tool. Eight of the twenty-four included studies were rated as ‘low risk of bias’, the other twelve as ‘some concerns’, and the remaining four as ‘high risk’. A non-randomized interventional study was assessed according to the ROBINS-I tool and ranked as ‘some concerns’. Risk of bias for all studies are presented in Figure 2, Figure 3 and Figure 4.

### 3.4. Preclinical Studies

A total of 19 in vivo studies were identified (Supplementary Material File S2).

#### 3.4.1. Preclinical Studies—Effect of Celecoxib on Depression and Mania-Like Symptoms in Animal Models

In all preclinical studies (17/17, 100%), regardless of the depression model, antidepressant effect of celecoxib was reported. Most included reports have pointed to the antidepressant effect of celecoxib in monotherapy [72,73,74,75,76,77,78,79,80,81,82,83,84,85,86,87]. Interestingly, two studies indicated that celecoxib might enhance the antidepressant effects of fluoxetine and bupropion [75,88]. In nine studies, celecoxib alone or with co-administration of antidepressants improved behavioral despair in forced swimming test (FST) [74,75,76,80,81,82,84,85,86] and in four studies in tail suspension test (TST) [75,76,78,83]. In eight studies, a positive effect on anhedonia as measured by the sucrose preference test (SPT) was found [73,76,77,81,82,84,85,86]. Four studies showed reduced anxiety and increased locomotor activity in open field test (OFT) [72,73,74,87] and one in evaluated plus maze (EPM) [76]. However, some studies produced ambiguous results. One study showed that celecoxib was effective for only five minutes and then did not show any further effect [72]. According to Alboni’s study, celecoxib partially restored stress-induced escape deficits when combined with fluoxetine. However, full reversal of the deficit was not achieved [88].

The models used to assess the influence of celecoxib on anxiety or depressive symptoms were as follows: stress-induced depressive-like behavior [73,77,78,81,84,86,88], induced by inflammation [74,79,82,85], diet-induced [76], different disease models [75,80,83], and bulbectomy [72,87].

Studies have used a dose in the range of 2–50 mg/kg/day, with most studies examining the effects of a dose of 15–30 mg/kg/day. The duration of celecoxib administration ranged from a single dose to 5 weeks, with an average of 17 days.

We identified two studies that evaluated the effects of celecoxib on mania-like symptoms in an animal model [89,90]. Both were conducted by the same research group, using a d-AMPH-induced mania model and administering 20 mg/kg/day celecoxib p.o. for 7 days. In both studies, celecoxib and low-dose lithium co-administered successfully abrogated the d-AMPH effect in open field test (OFT). Separate drug administration did not produce this effect.

#### 3.4.2. Preclinical Studies—Safety of Celecoxib in Rodents

No adverse effects related to celecoxib administration were reported in the preclinical studies analyzed. There was also no association of drug dose, route of administration, or time of administration with adverse effects.

#### 3.4.3. Preclinical Studies—Effect of Celecoxib on Inflammatory Markers

The effect of celecoxib on central or peripheral inflammatory markers in depression models was studied in eight papers. As a result of celecoxib administration, elevated central brain levels of PGE2 observed in depression models were decreased [73,81,87]. The treatment normalized brain levels of IL-1β [72,75,81,83], TNFα [72,81], and IFNγ [81] which were higher in depression models. Further, the levels of IL-10 were lower in the hypothalamus and higher in the prefrontal cortex in a group of rodents taking celecoxib [72]. In serum, celecoxib reduced IL-1 β and PGE2 concentration and blocked the elevation of corticosterone levels in one study [87], but no effect on peripheral blood cytokines was observed in another [72]. One study evaluating the effect of celecoxib administration on BDNF concentrations failed to find an association [75]. Celecoxib has also been found to attenuate reduction of NGF expression in the hippocampus [87] and affect neuroinflammation by inhibiting microglia activation [81,83,84].

Only one study evaluated changes in immune parameters in a mania model. Co-administration of celecoxib and lithium (24 mg/kg/day) reversed increased IL-4 in the frontal cortex, TNFα in the striatum, and IL-10 in the serum [89].

### 3.5. Clinical Studies

We identified 25 reports which concerned 17 trials; 16 of them were randomized controlled trials and 1 open-label clinical trial. Eight papers were secondary analyses including primary trials. Ten studies focused on the patient population with depression (Table 2), and 12 studies focused on bipolar disorder (n = 9—bipolar depression, n = 3—mania) (Table 3 and Table 4). Three studies were aimed at affective symptoms in somatic disorders (Table 5).

#### 3.5.1. Clinical Studies—Effectiveness of Celecoxib in Major Depression

Eight studies evaluated the efficacy of additional celecoxib therapy. Seven of them were double-blind randomized controlled trials and one was open-label trial. The duration of studies varied from 6 to 8 weeks and the dose of celecoxib ranged from 200 to 400 mg/daily. Four of them (4/7, 57%) showed positive effects of celecoxib in clinical symptoms after the intervention at any checkpoint [50,51,52,53], but only three at the endpoint [50,51,52]. The main treatments in these studies were SSRI [50,51,53] or NRI [52]. In three studies, improvement was not observed [49,54,55]. Celecoxib was used as an add-on treatment in these studies along with sertraline [55], vortioxetine [54], or ECT [49].

Only studies assessed as ‘low risk of bias’ or ‘some concerns’ were included in the meta-analysis [50,51,52,54]. Three studies were excluded due to the assessment as being of high risk of bias [49,53,55] (Figure 2).

**Figure 5:**
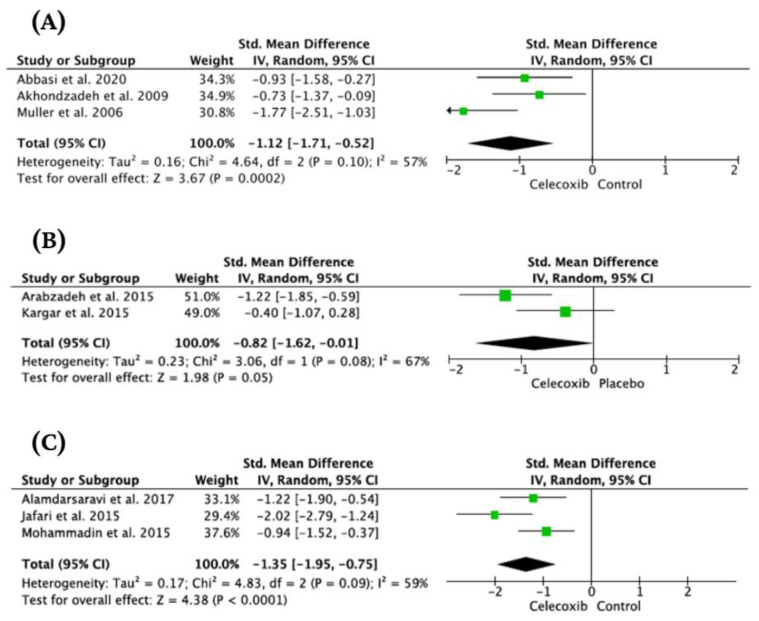
Forest plots for the effect of celecoxib as an added-on treatment in major depression (**A**) [50,51,52], mania (**B**) [59,60], and sole treatment of depressed patients with somatic comorbidity (**C**) [47,69,70].

The heterogeneity of studies evaluating the effect of celecoxib on major depression was substantial (I^2^ = 81%, Chi^2^ = 15.96, df = 3, Tau^2^ = 0.37, *p* < 0.001). The standardized mean difference (SMD) was −0.85 [95% CI: −1.52, −0.18]. Test for overall effect: Z = 2.5 (*p* = 0.01). However, we found discrepancies reflected in the considerable heterogeneity of analyzed studies. Specifically, we identified one significant outlier [54]. This study included mainly patients with TRD. Therefore, we performed a sensitivity analysis excluding the above study. It resulted in a substantial decrease in heterogeneity (I^2^ = 57%, Chi^2^ = 4.64, df = 2, Tau^2^ = 0.16, *p* = 0.10). SMD was −1.12 [−1.71, −0.52], (*p* = 0.0002) (Figure 5).

Visual evaluation of all funnel plots showed a symmetrical distribution, thus indicating the absence of publication bias.

As only one study with a TRD patient population was identified, the planned subgroup analysis could not be performed.

#### 3.5.2. Clinical Studies—Effectiveness of Celecoxib in Bipolar Disorder

##### Effectiveness of Celecoxib in Bipolar Depression

According to one of the three interventional studies (1/3, 33%), a significantly greater improvement in depressive symptoms was noted in the celecoxib group compared to placebo [56]. However, in one of two negative studies, celecoxib was superior to placebo in the assessment after 1 week of treatment, when the analysis included only the subjects who completed the full 6-week trial [57]. At the end of treatment (after 6 weeks), there were no statistically significant differences between the two groups. In turn, a study by Husain et al. found no advantage of celecoxib over placebo in any of the interim assessments nor at the end of treatment (after 12 weeks) [58].

The duration of studies varied from 6 to 12 weeks and the dose of celecoxib ranged from 200 to 400 mg/daily. Various mood stabilizers, antipsychotics, antidepressants, and benzodiazepines were used as the main treatments in two studies [57,58]; in one study, escitalopram was administered [56]. A meta-analysis and subgroup analysis were abandoned because raw data were not available in one study [56], and high risk of quality assessment was in another [57].

##### Effectiveness of Celecoxib in Mania

Celecoxib’s effect on mania symptoms has only been examined in two double-blind randomized controlled trials. Study durations and study samples were 6 weeks (N = 46) and 6 ECT sessions (N = 35). Both studies used celecoxib in a dose of 400 mg/day, however, they differed in the main treatment—sodium valproate in one [59] and ECT in the other [60]. Celecoxib augmentation was found to be superior to placebo only in one of these two studies (1/2, 50%) [59].

A substantial heterogeneity (I^2^ = 67%, Chi^2^ = 3.06, df = 1, Tau^2^ = 0.23, *p* = 0.08) was found during analysis. Calculated SMD was −0.82 [95% CI: −1.62, −0.01], (*p* = 0.05) (Figure 5).

#### 3.5.3. Clinical Studies—Safety of Celecoxib as an Added-on Treatment in Mood Disorders

Ten studies evaluated the incidence rates of adverse effects between celecoxib and placebo groups in major depression and bipolar disorder [48,50,51,52,53,54,56,57,58,59]. In all of the above studies no significant differences in the incidence rate of adverse effects between groups were observed, except skin and mucous membranes in one [54]. No serious adverse effects have been reported. Treatment with celecoxib did not affect cognition [48,54] or serum drug levels [51,52]. The acceptability of the treatment in both groups was similar in all studies.

#### 3.5.4. Clinical Studies—Effect of Celecoxib Treatment on Inflammatory Markers in Patients with Mood Disorders

A total of 15 studies evaluated parameters related to inflammation (Table 2, Table 3 and Table 4) [48,50,54,55,58,60,61,62,63,64,65,66,67,68]. The most commonly studied parameters were: CRP, kynurenine pathway metabolites, IL-2, IL-1β, TNF-α, MFI (macrophage migration inhibitory factor) (Table 6). Overall, none of the parameters studied were found to be significantly different in the celecoxib-treated group compared to the control group in more than one study. Both IL-6 [50] and TNF-alpha [48] and CRP [61] levels were significantly different in only a single study; the other studies did not confirm such regularity [47,54,55,58]. Table 6 presents the pooled analysis of the studies on a given blood inflammatory parameter.

#### 3.5.5. Clinical Studies—Effectiveness of Celecoxib in Depressed Patients with Somatic Comorbidity

Three studies evaluating celecoxib’s effect on depressive symptoms in somatic disorders were double-blind RCTs involving a patient population with mild to moderate depression. Two of them concerned cancer patients [46,69], and one concerned depressed patients diagnosed with brucellosis [70]. Study duration and sample ranged from 6 weeks (N = 40), and 6 weeks (N = 52) to 8 weeks (N = 40). Celecoxib was administered at a dose of 400 mg; the control group used placebo or diclofenac.

In all three studies (3/3, 100%), celecoxib group showed significantly greater improvement in HAMD-17 score compared to controls. The pooled effect of celecoxib in two studies with cancer patients was significant [46,69]. The heterogeneity of these studies was small (I^2^ = 0%, Chi^2^ = 0.37, df = 1, Tau^2^ = 0.00). The standardized mean difference (SMD) was −1.06 [−1.50, −0.62]. Test for overall effect: Z = 4.72 (*p* < 0.00001).

The heterogeneity of studies evaluating the effect of celecoxib on depression symptoms in patients with somatic disorders was substantial (I^2^ = 59%, Chi^2^ = 4.83, df = 2, Tau^2^ = 0.17, *p* = 0.09). The standardized mean difference (SMD) was −1.35 [95% CI: −1.95, −0.75]. Test for overall effect: Z = 4.38 (*p* < 0.0001) (Figure 5).

#### 3.5.6. Clinical Studies—Safety of Celecoxib in Depressed Patients with Somatic Comorbidity

No statistically significant differences in the frequency of adverse effects were observed between celecoxib and control groups [46,69,70].

#### 3.5.7. Clinical Studies—Effect of Celecoxib Treatment on Inflammatory Markers in Depressed Patients with Somatic Comorbidity

Inflammatory parameters were not investigated in any of the studies on depressed patients with somatic comorbidities.

## 4. Discussion

According to recent literature, inflammation may play an important role in the pathogenesis and course of mood disorders [91]. This prompts the consideration of anti-inflammatory treatment as a potential therapeutic approach. Therefore, we decided to summarize in a systematic way the current literature data on one of the agents with anti-inflammatory activity—celecoxib as a treatment for mood disorders. The main finding of this study is the efficacy of celecoxib at a dose of 400 mg used for 6 weeks as an add-on treatment in major depression and mania, as well as in depression with comorbid somatic conditions used as the sole antidepressant treatment.

The rationale behind the antidepressant’s effectiveness was found in both preclinical and clinical studies. The meta-analysis indicated that celecoxib is an effective add-on treatment for major depression. This result is consistent with the previous reviews [34,39,40,92]. Only one out of the high-quality studies we identified was not consistent with this result [54]. We found that this study was the only study conducted in patients with treatment-resistant depression (TRD) and showed no benefits from celecoxib use in this population. As one-third of MD patients may be refractory to treatment, and the search for effective augmentation strategies is still ongoing [93,94], further studies are needed to determine whether celecoxib has a beneficial effect in treatment-resistant populations as well.

Studies of celecoxib in bipolar depression have yielded somewhat different results and these are inconclusive. In only one of the identified studies, an improvement in depressive symptoms was noted [56]. Interestingly, the opposite was found in the studies with the MD population; this particular study involved the TRD BD population [56]. Further studies to resolve these ambiguities are needed. In contrast to bipolar depression, in the case of mania, the results were more conclusive, indicating that celecoxib was effective in this indication as an add-on treatment. Our findings are consistent with a previously conducted meta-analysis [41].

It is worth mentioning that the efficacy of celecoxib should be evaluated along with treatment adherence. However, this was reported in 7 out of 16 RCTs [47,53,56,57,58,69,71]. It included patient–staff interviews [53,71] and pill counts [47,56,57,58,69,71]. Additionally, in two studies, the serum level of the main treatment (reboxetine and fluoxetine) was measured [51,52]. Although none of the above RCTs reported poor adherence, it should be noted that in the remaining studies, it was not reported. This issue was partly addressed during the risk of bias assessment, however, we cannot entirely exclude that this might have affected some results.

The included trials ranged in duration from 6 to 12 weeks aiming to assess the efficacy of celecoxib in the short to medium term. However, anti-inflammatory agents may have long-term positive effects. In a recent review, low-dose aspirin was found to reduce the risk of reoccurrence of all affective episodes of bipolar disorder [95]. In this context, studies with long-term follow-up, targeting the assessment of recurrent affective episodes would be required to evaluate the potential efficacy of celecoxib in the treatment of relapses. Obviously, this is where the safety of such treatment comes into consideration, especially when considering long-term therapy. The safety of celecoxib in mood disorders has also been raised in this review. Several years ago, the FDA imposed a black box warning about the drug’s cardiovascular risk. This is particularly relevant to patients suffering from bipolar disorder who are at heightened risk of cardiovascular events, which remain a leading cause of death in this population [96,97,98]. However, based on the PRECISION study, the FDA has backed down on this warning in recent years. Celecoxib, at a dose of 2 × 100 mg, had the same effect on cardiovascular risk as other NSAIDs [99]. As a result of this review, we concluded that celecoxib could be used safely in mood disorders at a dose of 400 mg per day for 6–12 weeks. Furthermore, 400 mg/day was safe for patients with somatic conditions taking it for 6–8 weeks. Cardiovascular complications were not reported. However, the risk of cardiovascular complications may rise with an increasing dose and length of treatment, thus it has been recommended to use it for the shortest possible time and at the lowest effective daily dose (the maximum recommended daily dose is 400 mg for all indications). Furthermore, it is critical to avoid administering this medication to patients with contraindications, including those with hypersensitivity to the active ingredient, sulfonamides or other nonsteroidal anti-inflammatory drugs, active gastric or duodenal ulcer disease, or pregnant and breastfeeding patients. Long-term studies can be considered to determine whether celecoxib is effective in recurrent mood disorders. However, this would require an evaluation of a safe and effective dose for long-term use in this indication.

Celecoxib’s doses in mood disorders should be investigated. According to clinical studies, this drug provides greater pain relief and inflammation reduction at higher doses, but at the same time increases adverse effects. The majority of studies we found used a celecoxib dose of 400 mg, while two used 200–400 mg [58,71] and one used 200 mg [53]. Therefore, the dose–effect relationship cannot be concluded on this basis. To estimate this connection, further studies including various doses of celecoxib should be performed. It should be noted that, currently, the maximum dose recommended for all indications according to the Summary of Product Characteristics is 400 mg.

As a result of its anti-inflammatory and analgesic properties, celecoxib is often used to treat somatic diseases such as rheumatoid arthritis, osteoarthritis, and neuralgia [100,101,102]. According to our meta-analysis, celecoxib at a dose of 400 mg/d used for 6–8 weeks as the sole treatment in patients with the somatic disease and comorbid depression was significantly more effective in antidepressant efficacy than place and the comparator (diclofenac). Nevertheless, we identified only three studies involving patient populations with brucellosis, colorectal cancer, and breast cancer [47,69,70]. Although brucellosis is an infectious disease, neurobrucellosis can also clinically manifest as depression [103]. In light of the high incidence and mortality of different types of cancer in recent years, celecoxib’s efficacy in this patient population seems to be important information [104,105]. Patients with cancer are more likely to experience depression and chronic pain compared to the general population [106,107]. It has been shown that celecoxib is a good therapeutic option for reducing pain, as well as improving mood. An analysis of pooled data from five post approval trials also showed that this drug significantly reduced depressive symptoms in patients with osteoarthritis at a dose of 200 mg daily [108]. Considering that depression can accompany many diseases, it is important to study celecoxib’s use in patients with other somatic conditions, in particular when treatments are based primarily on pain management. Comparison with other commonly used NSAIDs, such as diclofenac, may also provide important information. Although celecoxib appears to improve depression symptoms in somatic patients, none of the papers we reviewed examined the drug’s association with inflammatory markers. As it seems that this could provide important data regarding a possible common underlying origin of both conditions, there is a need for further research on these issues in this group of patients.

The potential therapeutic effect of celecoxib is likely to be due to its ability to act via COX-2 and its effect on the arachidonic pathway [109]. Moreover, inhibition of this enzyme might directly affect the serotonergic system in the central nervous system [110]. Celecoxib inhibits COX-2 selectively, therefore PEG2 levels are decreased and the balance of pro-inflammatory and anti-inflammatory cytokines is altered. There are various pathways through which inflammation can be modulated, affecting the concentrations of inflammatory and neurotrophic markers. IL-1, TNFα, IFNγ, NGF, or microglia activation in depression, as well as IL-4, TNFα, and IL-10 in mania, were normalized by celecoxib in preclinical studies. Nevertheless, the results of preclinical studies have not been confirmed in clinical trials. Celecoxib treatment only improved peripheral IL-6 levels in depression [50] and TNF-alpha levels in mania [48], but these are only single clinical studies on these markers. There were negative results for other substances (such as kynurenine pathway metabolites) or unclear results (such as CRP). Our findings from preclinical studies can be used to identify future research directions in clinical trials. For instance, preclinical studies have demonstrated positive results regarding central IFNγ and NGF for depression [81,87], as well as peripheral IL-10 for mania [89]. To confirm these findings, translational studies would be needed. Attwells et al. proposed a new approach to predicting the effects of celecoxib treatment in their open-label study using PET method [71]. Celecoxib was more effective in treating patients with severe gliosis determined by using translocator protein total distribution volume in the anterior cingulate cortex and prefrontal cortex. There is a need for further randomized controlled studies to confirm this method’s effectiveness in predicting anti-inflammatory responses. However, mood disorders, particularly MDD, are very complicated and varied conditions. Although genetic, neurobiological, or environmental factors are known to have a significant influence, the pathophysiology of this disorder is not entirely understood, as we previously mentioned. MDD patients’ immunological states are inconsistent with notable interindividual variations. This necessitates careful interpretation of the outcomes of numerous therapies on this patient group [111]. Finding markers that identify MDD patients who respond better to a specific adjunctive therapy prove crucial for personalizing therapy. However, the results of the studies in this field are still unclear. The development of predictors of treatment response should be conducted to identify patients with inflammatory phenotypes who will benefit from celecoxib augmentation.

Finally, it is important to consider celecoxib’s pharmacokinetic interaction with antidepressants as another possible mechanism explaining its positive effect on depression symptoms. Most antidepressants are metabolized by two major metabolic enzyme systems: cytochrome P450 (CYP) or UDP- glucuronosyltransferases (UGT) [112]. Celecoxib is mainly metabolized by CYP2C9 in the liver. Among its pharmacokinetic interactions, inhibition of CYP2D6 and inhibition of CYP2C19 contribute to the suppression of the metabolism of substances catalyzed by these enzymes. Among these compounds are antidepressants, including those identified in our review: CYP2D6 metabolizes fluoxetine and vortioxetine, while CYP2C19 metabolizes sertraline. In the only study we found which assessed fluoxetine levels in both celecoxib and control groups, no difference was found between them [51]. It is further surprising that there is no clear drug–drug association since fluoxetine is also metabolized by CYP2C9, which is the main metabolic pathway for celecoxib [112]. Therefore, the pharmacokinetic responses that might be expected were not observed. Furthermore, there was no difference in reboxetine concentrations between celecoxib and control groups [52]. This finding is, however, backed up by the fact that the drug is not metabolized by CYP2D6. As a result of these findings, celecoxib did not elevate antidepressant levels. There is also evidence that anti-inflammatory drugs that affect COX enzymes, such as aspirin, can also be used as an effective augmentation method, although they affect CYP in very different ways—they do not affect CYP2D6 and instead induce CYP2C19 activity [113]. Evaluation of the serum concentration of celecoxib and the main antidepressant treatment will be critical in future studies to confirm and clarify these findings.

Several studies have demonstrated that inflammation pathways play a critical role in improving symptoms of mood disorders. According to a recent systematic review, aspirin, which inhibits both COX-1 and COX-2, is an effective and safe adjunctive treatment option for MDD and BD in adults [95]. Other anti-inflammatory drugs, such as minocycline or N-acetylcysteine, are also beneficial to patients with mood disorders [114,115]. Additionally, in some cases, combined anti-inflammatory treatments may be effective. We identified one study in which minocycline + celecoxib was no better than placebo [58], however, in another NSAID study with aspirin, it was made more effective when combined with N-acetylcysteine [116]. The benefits of possible combinations have been assessed in further studies.

## 5. Conclusions

This study suggests the antidepressant efficacy of celecoxib at a dose of 400 mg used for 6 weeks as an add-on treatment for major depression and mania. Furthermore, celecoxib in the above dosage used as sole treatment was also effective in reducing depressive symptoms in depressed patients with somatic comorbidity. No conclusive evidence on the antidepressant efficacy of celecoxib in bipolar depression was found. Celecoxib at a dose of 400 mg/d used for up to 12 weeks appears to be a safe treatment for patients with mood disorders. Although an association between celecoxib response and inflammatory parameters has been found in preclinical studies, this has not been confirmed in clinical trials. Therefore, based on the available studies to date, it is not possible to identify a marker of inflammation in the given types of depression and affective episodes in stratifying patients on this basis.

Further high-quality RCTs are needed to evaluate celecoxib efficacy in bipolar depression. Other identified research gaps include evaluating the efficacy of celecoxib in treatment-resistant depression (TRD), the efficacy in preventing relapse in recurrent mood disorders, and finally, the association of celecoxib treatment with inflammatory cytokines, particularly in patients with comorbid somatic disorders.

## Figures and Tables

**Table 1:** PICO framework for a different design of studies.

	Preclinical Studies	Observational Studies	Interventional Studies
Patients/Subjects	Studies on depression or mania animal models. Animals may or may not be treated with antidepressants or mood stabilizers.	Patients receiving celecoxib with or without a diagnosis of major depression (MD) or bipolar disorder (BD), under 65 years of age and over 18 years of age.	Patients diagnosed with MD or BD, or without diagnoses as above but with affective symptoms under 65 years of age and over 18 years of age.
Intervention	Use celecoxib alone or as an add-on treatment.	Patients receiving celecoxib.	Patients treated in combination with celecoxib.
Comparison	No use of celecoxib.	Not receiving celecoxib.	No use of celecoxib; placebo.
Outcome	Impact on: (1) behavioral tests of assessment despair—FST, TST; anhedonia—SPT; anxiety and locomotor activity—OFT, LA, EPM; escape test; fear—HC, FCP; (2) adverse effects; (3) inflammatory markers (PGE2, interleukins, neurotrophic factors, microglial activation markers; kynurenine metabolites).	Episodes of depression, and mania. Hazard ratio (HR); Incident density (IR); Odds ratio (OD). Inflammatory markers (PGE2, interleukins, neurotrophic factors, microglial activation markers; kynurenine metabolites).	Impact on: (1) effectiveness in relieving symptoms of depression and mania—clinical scales (HAMD-17, HAMD-21, MADRS, YMRS); response rate (50% improvement in any clinical scale); remission rate (≤7 in HAMD-17, MADRS for depressive episode and ≤7 in YMRS for manic episode); (2) safety measured by adverse effects (difference between intervention and control groups) and acceptability (dropping out due to any reason); (3) inflammatory markers (PGE2, interleukins, neurotrophic factors, microglial activation markers; kynurenine metabolites).

FST—forced swimming test, TST—tail suspension test, SPT—sucrose preference test, OFT—open field test, LA—locomotor activity, EPM—evaluated plus maze, HC—home cage test, FCP—fear conditioning paradigm, HAMD-17—Hamilton Depression Rating Scale, 17 items, MADRS—Montgomery–Asberg Depression Rating Scale, YMRS—Young Mania Rating Scale.

**Table 2:** Clinical studies on celecoxib for major depression (n = 10).

Authors, Year, Country	Study Design; Duration	Sample Size, Population	Treatment	Results: (1) Efficacy; (2) Adverse Effects; (3) Inflammatory Markers
Muller et al. 2006, Germany [52]	Randomized, double-blind, placebo-controlled two-arm trial; 6 weeks	N = 40 MD (296.2–296.3) Aged 23–65 HAMD-17: ranged 15–48 points	Celecoxib 400 mg/day + Reboxetine (4–10 mg) vs. Placebo + Reboxetine (4–10 mg)	(1) CEL group showed significantly greater improvement in HAMD-17 scores versus placebo group (*p* = 0.035); no statistically significant differences in responder and remitter rates between groups were observed; (2) No adverse effects that have been attributed to the CEL administration were observed; in both groups, reasons leading to drop-out were typical for noradrenergic drugs; no difference in plasma level of reboxetine was observed (*p* = 0.17), therefore noncompliance and drug–drug interactions were possible to exclude; 10 patients from CEL group and 12 patients from placebo group dropped out from the study
Akhondzadeh et al. 2009, Iran [51]	Randomized, double-blind, placebo-controlled two-arm trial; 6 weeks	N = 40 MD Aged 24–46 HAMD-17 ≥ 18 points	Celecoxib 400 mg/day + Fluoxetine 40 mg/day vs. Placebo + Fluoxetine 40 mg/day	(1) CEL group showed significantly greater improvement in HAMD-17 versus placebo at the endpoint, *p* = 0.001; responders: 90% vs. 50%, *p* = 0.01; remitters: 35% vs. 5%, *p* = 0.04; (2) No statistically significant differences in side effects between groups were observed; no statistically significant differences in plasma level of fluoxetine were observed, therefore noncompliance and drug–drug interactions were possible to exclude; 1 patient from CEL group and 2 patients from placebo group dropped out from the study
Abbasi et al. 2012, Iran [50]	Randomized, double-blind, placebo-controlled two-arm trial; 6 weeks	N = 40 MD Aged 18–50 HAMD-17 ≥ 18 points	Celecoxib 400 mg/day + Sertraline 200 mg/day vs. Placebo + Sertraline 200 mg/day	(1) CEL group showed significantly greater improvement in HAMD-17 versus placebo in endpoint, *p* < 0.001; responders: 95% vs. 50%, *p* = 0.003; remitters: 35% vs. 5%, *p* = 0.04; (2) No statistically significant differences in side effects between groups were observed; 1 patient from CEL group and 2 patients from placebo group dropped out from the study; (3) Il-6 significant reduction was observed in both groups, however, in celecoxib group, reduction was greater (*p* < 0.001); reduction correlated with HAMD-17 score; responders and remitters had greater reduction of Il-6
Majd et al. 2015, Iran [53]	Randomized, double-blind, placebo-controlled two-arm trial; 6 weeks	N = 30 MD first episode (drug-I) Aged 18–50 HAMD-17: 18–36 points	Celecoxib 200 mg/day + Sertraline 25–100 mg/day vs. Placebo + Sertraline 25–100 mg/day	(1) CEL group showed significantly greater improvement in HAMD-17 versus placebo after 4 weeks, (*p* < 0.05), but not at the endpoint; responders: 57% vs. 11%, *p* < 0.05 after 4 weeks, at the endpoint: 100% vs. 77.7%, *p* = 0.14; remitters: no information after 4 weeks, at the endpoint: 57% vs. 11%, *p* < 0.05; adherence was assessed each week via phone; (2) No statistically significant differences in side effects between groups were observed; 1 patient from CEL group and 6 patients from placebo group dropped out from the study
Banaha et al. 2019, Iran [49]	Randomized, double-blind, placebo-controlled two-arm trial; 6 sessions of ECT	N = 20 MD Aged 20–75 ECT was indicated	Celecoxib 400 mg/day + ECT vs. Placebo + ECT	(1) No significant difference was observed between celecoxib group (400 mg/day) versus placebo in HAMD-17 scale throughout 6 ECT sessions, *p* = 0.39; (2) No statistically significant differences in side effects between groups were observed; CEL did not affect cognition in positive or negative way—no statistically significant differences in WMS-III, MMSE, SC between groups were observed
Simon et al. 2021, Germany [55]	Randomized, double-blind, placebo-controlled two-arm trial; 6 weeks	N = 43 MD Aged 18–60 MADRS ≥ 20	Celecoxib 400 mg/day + Sertraline 50–100 mg/day	(1) No statistically significant difference was observed between CEL group versus placebo in MADRS scale after 6 weeks; no statistically significant differences in responder and remitter rates between groups were observed; (2) 0 patients from CEL group and 6 patients from placebo group dropped out from the study; (3) Clear pattern to MIT, neopterin, TNF-α was not observed
Baune et al. 2021, Australia [54]	Randomized, double-blind, placebo-controlled two-arm trial; 6 weeks	N = 119 MD; 76% treatment resistant Aged median: 47 MADRS ≥ 20	Celecoxib 400 mg/day + Vortioxetine	(1) No significant difference was observed between CEL group (400 mg/day) versus placebo in MADRS scale after 6 weeks, *p* > 0.05; no statistically significant differences in responder and remitter rates between groups were observed; (2) No statistically significant differences in side effects between groups were observed except skin or mucous membranes (more in the CEL group, *p* = 0.006). CEL did not affect cognition; 10 patients from CEL group and 10 patients from placebo group dropped out from the study; (3) hsCRP did not predict a better response to CEL augmentation—no statistically significant differences between treatment groups were observed for individuals with higher hsCRP
Attwells et al. 2020, Canada [71]	Open-label trial; 8 weeks	N = 41 MD treatment resistant Aged 18–58 HAMD-17 ≥ 9	Celecoxib 200–400 mg/day	(1) 6 participants were responders and 3 were remitters at the endpoint; compliance was assessed through patient–staff interviews and pill count; (3) Higher TSPO V_T_ in ACC and PFC measured with PET was related to the reduction on the HAMD-17 scale
Musil et al. 2011, Germany [62]	Secondary analysis of Muller et al. 2006 study; 5 weeks	N = 32 and 20 healthy controls MD Aged 25–65 HAMD-17 15–48 points	Celecoxib 400 mg/day + Reboxetine 4–10 mg/day vs. Placebo + Reboxetine 4–10 mg/day	(3) No statistically significant differences in MIF, TGF-β and sCD14 levels were observed between CEL and placebo group
Krause et al. 2017, Germany [63]	Secondary analysis of Muller et al. 2006 study; 5 weeks	N = 32 and 20 healthy controls MD Aged 23–63 HAMD-17: 15–38	Celecoxib 400 mg/day + Reboxetine 4–10 mg/day vs. Placebo + Reboxetine 4–10 mg/day	(3) Tryptophan metabolites did not differ significantly between the CEL and control groups after 6 weeks of treatment; higher KYN/TRP was predictive for remission to antidepressants with or without CEL

MD—major depression, CEL—celecoxib, HAMD-17—Hamilton Depression Rating Scale, 17 items, ECT—electroconvulsive therapy, WMS-III—Wechsler Mental Scale III, MMSE—Mini-Mental Scale Examination, SC—Stroop Color test, MADRS—Montgomery–Asberg Depression Scale, hsCRP—high sensitivity C-reactive protein, MIT—macrophage migration inhibitory factor, TNFα—tumor necrosis factor α, TSPO V_T_—translocator protein total distribution volume, PET—positron emission tomography, ACC—anterior cingulate cortex, PFC—prefrontal cortex, KYN/TRP—kynurenine/tryptophan ratio.

**Table 3:** Clinical studies on celecoxib for bipolar depression (n = 9).

Authors, Year, Country	Study Design; Duration	Sample Size, Population	Treatment	Results: (1) Efficacy; (2) Adverse Effects; (3) Inflammatory Markers
Nery et al. 2008, USA [57]	Randomized, double-blind, placebo-controlled two-arm trial; 6 weeks	N = 28 BD patients in depressive or mixed episode Aged 22–61 HAMD-21 ≥ 18	Celecoxib 400 mg/day vs. Placebo (mood stabilizers, antipsychotics, antidepressants, benzodiazepines)	(1) No statistically significant difference was observed between CEL group versus placebo in HAMD-21 scale after 6 weeks; CEL was superior to placebo in the assessment after 1 week of treatment, when the analysis included only the subjects who completed the full 6-week trial; compliance was assessed by pill counting; (2) No statistically significant difference in the prevalence of adverse effects between groups; 2 patients from CEL group dropped out from the study (because of rush) and 3 patients from placebo group dropped out from the study
Halaris et al. 2020, USA [56]	Randomized, double-blind, placebo-controlled two-arm trial; 10 weeks	N = 47 BD treatment-resistant patients with depressive episode Aged 20–65 HAMD-17 ≥ 18	Celecoxib 400 mg/day + Escitalopram 20 mg/day vs. Placebo + Escitalopram 20 mg/day	(1) CEL group scored significantly lower in HAMD-17 scale compared to placebo group at the endpoint (*p* = 0.002); as fast as after 1 week of the trial scores on the HAMD-17, HAMD-7 and HAMD-21 were significantly lower in the CEL group as compared to placebo (all *p* < 0.005), responders: 78% vs. 45%, *p* = 0.021 remitters: 63% vs. 10%, *p* < 0.0005; compliance was assessed by pill counting; (2) No significant difference in the occurrences of side effects between groups were observed; 8 patients from CEL group and 10 patients from placebo group dropped out from the study
Husain et al. 2020, Pakistan [58]	Randomized, double-blind, placebo-controlled four- arm trial; 12 weeks	N = 266 BD patients with bipolar depression Aged 18–65 HAMD-17 ≥ 18	Celecoxib 200–400 mg/day + Placebo vs. Celecoxib 200–400 mg/day + Minocycline 100–200 mg/day vs. Minocycline 100–200 mg/day + Placebo vs. Placebo + Placebo (mood stabilizers, antipsychotics, antidepressants, benzodiazepines)	(1) CEL group did not differ significantly in HAMD-17 scores compared to placebo group at the endpoint (*p* = 0.443); responders: 54% vs. 58%, *p* = 0.505 remitters: 38% vs. 24%, *p* = 0.036; compliance was assessed by pill counting; (2) No statistically significant difference in the incidence rate of side effects was observed; 7 patients from CEL group and 10 patients from placebo group dropped out from the study; (3) The effects of celecoxib were not moderated by CRP level (*p* = 0.28) or WBC (*p* = 0.28)
Edberg et al. 2018, USA [61]	Secondary analysis of Halaris et al. study; 10 weeks	N = 47 BD treatment-resistant patients with depressive episode and N = 35 healthy controls Aged 20–65 HAMD-17 ≥ 18	Celecoxib 400 mg/day + Escitalopram 20 mg/day vs. Placebo + Escitalopram 20 mg/day	(3) CRP was significantly decreased in CEL group vs. placebo by week 8 (*p* = 0.0033)
Murata et al. 2019, USA [64]	Secondary analysis of Halaris et al. study; 10 weeks	N = 47 BD treatment-resistant patients with depressive episode and N = 35 healthy controls Aged 20–65 HAMD-17 ≥ 18	Celecoxib 400 mg/day + Escitalopram 20 mg/day vs. Placebo + Escitalopram 20 mg/day	(3) Clinical response to CEL augmentation was not associated with altered neurotoxic or neuroprotective measured by kynurenine pathway metabolites;
Edberg et al. 2020, USA [66]	Secondary analysis of Halaris et al. study; 10 weeks	N = 47 BD treatment-resistant patients with depressive episode and N = 35 healthy controls Aged 20–65 HAMD-17 ≥ 18	Celecoxib 400 mg/day + Escitalopram 20 mg/day vs. Placebo + Escitalopram 20 mg/day	(3) MCP-1 were not statistically different between CEL and placebo group by week 8; MCP-1 was positively correlated with anti-inflammatory analytes in CEL group (IL-4, IL-6, IL-10)
Murata et al. 2020, USA [65]	Secondary analysis of Halaris et al. study; 10 weeks	N = 47 BD treatment-resistant patients with depressive episode and N = 43 healthy controls Aged 20–65 HAMD-17 ≥ 18	Celecoxib 400 mg/day + Escitalopram 20 mg/day vs. Placebo + Escitalopram 20 mg/day	(3) There were no statistical differences in the IL-1β or KYN/TRP levels after treatment between placebo and escitalopram + CEL group; responders/non-responders (*p* = 0.239, and *p* = 0.146, respectively); By week 8, responders showed a downtrend in IL-1β compared to non-responders in the escitalopram + CEL treatment arm
Castillo et al. 2020, USA [68]	Secondary analysis of Halaris et al. study; 10 weeks	N = 47 BD treatment-resistant patients with depressive episode and N = 32 healthy controls Aged 20–65 HAMD-17 ≥ 18	Celecoxib 400 mg/day + Escitalopram 20 mg/day vs. Placebo + Escitalopram 20 mg/day	(3) At all timepoints, patients receiving CEL had comparable VEGF values (mean = 16.10, SE = 1.43) to those receiving placebo (mean = 14.51, SE = 1.75, *p* = 0.49)
Murata et al. 2021, USA [67]	Secondary analysis of Halaris et al. study; 10 weeks	N = 47 BD treatment-resistant patients with depressive episode and N = 43 healthy controls Aged 20–65 HAMD-17 ≥ 18	Celecoxib 400 mg/day + Escitalopram 20 mg/day vs. Placebo + Escitalopram 20 mg/day	(3) The absence of interaction effects between treatment arm and baseline salivary cortisol suggests a generalized effect of hypercortisolemia on treatment response across escitalopram + placebo and escitalopram + CEL treatments

BD—bipolar disorder, CEL—celecoxib, HAMD-21—Hamilton Depression Rating Scale, 21 items, HAMD-17—Hamilton Depression Rating Scale 17 items, HAMD-7—Hamilton Depression Rating, 7 items, CRP—C-reactive protein, WBC—white blood cells, M–P-1—monocyte chemoattractant protein-1, IL—interleukin, KYN/TRP—kynurenine/tryptophan ratio, MCP-1—monocyte chemoattractant protein-1, VEGF—vascular endothelial growth factor, SE—standard error.

**Table 4:** Clinical studies on celecoxib for mania (n = 3).

Authors, Year, Country	Study Design; Duration	Sample Size, Population	Treatment	Results: (1) Efficacy; (2) Adverse Effects; (3) Inflammatory Markers
Arabzadeh et al. 2015, Iran [59]	Randomized, double-blind, placebo-controlled two-arm trial; 6 weeks	N = 46 BD patients in manic episode Aged 18–50 YMRS ≥ 20	Celecoxib 400 mg/day + Sodium Valproate 600–800 mg/day vs. Placebo + Sodium Valproate 600–800 mg/day	(1) CEL group showed significantly greater improvement on YMRS versus placebo group after 6 weeks (*p* < 0.001); responders: 100% vs. 82.6%, *p* = 0.11; remitters at the endpoint: 87% vs. 43.5%, *p* = 0.005; (2) No statistically significant differences in the incidence rate of side effects between groups were observed
Kargar et al. 2014, Iran [48]	Randomized, double-blind, placebo-controlled two-arm trial; 6 sessions of ECT	N = 48 BD patients with manic episode (72% in CEL group and 78% in PLA group), depressive episode (12%/9%) or mixed (16%/13%) Aged 17–70 Indications to ECT	Celecoxib 400 mg/day + ECT vs. Placebo + ECT	(3) CEL group had significantly greater reduction of TNFα after 6 ECT sessions versus placebo group (*p* = 0.04); no statistically significant differences between groups in reduction of IL-1β, IL-6, CRP were observed
Kargar et al. 2015, Iran [60]	Randomized, double-blind, placebo-controlled two-arm trial; 6 sessions of ECT	N = 35 BD patients in manic episode Aged 17–65 YMRS ≥ 20	Celecoxib 400 mg/day + ECT vs. Placebo + ECT	(1) No significant difference was observed between CEL group versus placebo in YMRS scale after 6 ECT sessions, *p* = 0.397; no statistically significant differences in responder and remitter rates between groups were observed; (3) Serum BDNF was not significantly different between groups after treatment (*p* = 0.16)

BD—bipolar disorder, CEL—celecoxib, YMRS—Young Mania Rating Scale, ECT—electroconvulsive therapy, TNFα—tumor necrosis factor α, IL—interleukin, hsCRP—high sensitivity C-reactive protein, BDNF—brain-derived neurotrophic factor.

**Table 5:** Clinical studies on celecoxib for depression in course of somatic diseases (n = 3).

Authors, Year, Country	Study Design; Duration	Sample Size, Population	Treatment	Results: (1) Efficacy; (2) Adverse Effects; (3) Inflammatory Markers
Mohammadinejad et al. 2015, Iran [47]	Randomized, double-blind, placebo-controlled two-arm trial; 6 weeks	N = 52 Breast cancer patients who needed analgesics Aged 18–70 HAMD-17 < =18—mild to moderate depression	Celecoxib 400 mg/day vs. Diclofenac 100 mg/day	(1) CEL group showed significantly greater improvement in HAMD-17 versus diclofenac at the endpoint, *p* = 0.002; compliance was assessed by capsule counting; (2) No statistically significant differences in side effects between groups were observed
Jafari et al. 2015, Iran [70]	Randomized, double-blind, placebo-controlled two-arm trial; 8 weeks	N = 40 Depression due to brucellosis Aged 18–50 HAMD-17 < 19—mild to moderate depression	Celecoxib 400 mg/day vs. Placebo and antibiotics therapy in both groups	(1) CEL group showed significantly greater improvement in HAMD-17 versus placebo at the endpoint, *p* < 0.001; responders (50% imp.): 50% vs. 0%, *p* < 0.001; none experienced remission in both groups; (2) No statistically significant differences in side effects between groups were observed
Alamdarsaravi et al. 2017, Iran [69]	Randomized, double-blind, placebo-controlled two-arm trial; 6 weeks	N = 40 Colorectal cancer Aged 18–65 HAMD-17: 8–18— mild to moderate depression	Celecoxib 400 mg/day vs. Placebo	(1) CEL group showed significantly greater improvement in HAMD-17 versus placebo at the endpoint, *p* = 0.003; responders: 75% vs. 25%, *p* = 0.004; remitters: 45% vs. 25%, *p* = 0.32; compliance was assessed by capsule counting; (2) No statistically significant differences in side effects between groups were observed; 1 patient from CEL group and 2 patients from placebo group dropped out from the study

HAMD-17—Hamilton Depression Rating Scale, 17 items.

**Table 6:** Effect of celecoxib on blood inflammatory parameters in patients with mood disorders.

Inflammatory Marker	Studies Reporting on Given Variable—(n/N) (n—Number of Studies Where Variable Was Significantly Different versus Control at the Endpoint; Effect of Celecoxib Was Moderate by Variable; Variable Predict Response to Celecoxib, not Placebo/N—Number of all Studies Evaluating Given Variable)		
	Patients with Major Depression	Patients with Bipolar Depression	Patients with Mania
Interleukin-6 (IL-6) [48,50]	1/1	-	0/1 *
C-reactive protein (CRP) [48,54,58,61]	0/1	1/2	0/1 *
Interleukin 1β (IL-1β) [48,65]	-	0/1	0/1 *
Macrophage Migration Inhibitory Factor (MIF) [55,62]	0/2	-	-
TGF-β [62]	0/1	-	-
TNF-α [48,55]	0/1	-	1/1 *
BDNF [60]	-	-	0/1
sCD14 [62]	0/1	-	-
Neopterin [55]	0/1	-	-
Kynurenine pathway metabolites [63,64,65]	0/1	0/2	-
Monocyte chemoattractant protein-1 [66]	-	0/1	-
Salivary cortisol [67]	-	0/1	-
Vascular Endothelial Growth Factor (VEGF) [68]	-	0/1	-

* In this study, patients were in various affective state, most with mania.

## Data Availability

The data presented in this study are available upon request from the corresponding author. The data are not publicly available due to privacy or ethical concerns.

## Supplementary Materials

The following supporting information can be downloaded at: https://www.mdpi.com/article/10.3390/jcm12103497/s1. Supplementary Material File S1: Full search strategy for each database and registry. Supplementary Material File S2: In vivo studies in animal models of depression and mania (n = 19).
