# Supplementary material for: Celecoxib for Mood Disorders: A Systematic Review and Meta-Analysis of Randomized Controlled Trials
Source: J Clin Med. 2023 May 16;12(10):3497. doi: 10.3390/jcm12103497 (PMC10218898; doi:10.3390/jcm12103497)
Supplement: Supplementary file 1 [file jcm-12-03497-s001.zip › Supplementary Material File S1.pdf]

Supplementary Materials – Supplement to the description of the methodology

1. *Search strategy*

Table S1. Full search strategy for each database and registry.

Database/registry	Query	Filters and limits	Number of reviewers	Date of search	Results
PubMed	("Bipolar Disorder"[Mesh] OR "bipolar depression" OR "Mania"[Mesh] OR "hypomania" OR "mixed episode" OR "major depression" OR "depression" OR "Mood Disorders"[Mesh]) AND ("Celecoxib" OR "celebrex" OR "4-(5-(4-methylphenyl)- 3-(trifluoro methyl)-1H-pyrazol-1-yl) benzenesulfonamide")	Not applied	2	November 28, 2022	144
Scopus	("bipolar disorder" OR "bipolar depression" OR "mania" OR "hypomania" OR "mixed episode" OR "major depression" OR "depression" OR "mood disorders") AND ("celecoxib" OR "celebrex" OR "4-(5-(4-methylphenyl)- 3-(trifluoro methyl)-1H-pyrazol-1-yl) benzenesulfonamide")	Not applied	2	November 28, 2022	1224
Web of Science	("bipolar disorder" OR "bipolar depression" OR "mania" OR "hypomania" OR "mixed episode" OR "major depression" OR "depression" OR "mood disorders") AND ("celecoxib" OR "celebrex" OR "4-(5-(4-methylphenyl)- 3-(trifluoro methyl)-1H-pyrazol-1-yl) benzenesulfonamide")	Not applied	2	November 28, 2022	272
ClinicalTrials.gov	Condition or disease: "bipolar disorder" OR "bipolar depression" OR "mania" OR "hypomania" OR "mixed episode" OR "major depression" OR "depression" OR "mood disorders"<br>Other terms: celecoxib OR celebrex OR 4-(5-(4-methylphenyl)- 3-(trifluoro methyl)-1H-pyrazol-1-yl) benzenesulfonamide	Not applied	2	November 28, 2022	8

2. *Quality assesment*

Risk of bias of clinical trials was conducted in accordance with the Cochrane Collaboration guidelines [1]. Rob2 (Revised Cochrane risk of bias tool for randomized trials) was used to evaluate randomized clinical trials (RCTs) based on the following domains: randomization process, deviation from intended intervention, missing outcome data, measurement of the outcome, and selection of the reported results [2]. For interventional studies without randomization, ROBINS-I (The risk of bias in Non-randomized studies – of Interventions assessment tool) was applied to assess the following domains: confounding, selection of participants, classification of intervention, deviation from intended intervention, missing data, measurements of outcomes, and selection of reported results [3]. Risk of bias assessment was carried out for each study individually, and the results were presented separately for each study and cumulatively for each domain. The Robvis tool was used for visualization [4]. At least two authors independently assessed the paper, establishing consensus when disagreements arose.

3. *Synthesis and Analysis*

Search results from Mendeley Desktop have been transferred to Review Manager (RevMan5 version 5.4; Cochrane Collaboration). Studies were separated into two categories based on preclinical or clinical character. In preclinical studies, efficacy, safety, and inflammatory parameters were evaluated according to the model: depression or mania. According to the patient population, clinical trials were divided into groups according to the diagnosis: MD, BD

(mania/mixed state or depression), and non-psychiatric disorders. Efficacy, safety, and inflammatory parameters were analyzed in clinical studies.

Continuous outcomes were pooled as standardized mean difference (SMD). Heterogeneity was evaluated visually on the Forest plot and statistically using the Chi2, I2, and Tau2. Thresholds from Cochrane Collaboration were consistent with interpretation of heterogeneity: 0-40% might be not important; 30-60% may represent moderate heterogeneity; 50-90% may represent substantial heterogeneity, and 75-100% high level of heterogeneity [1]. Whenever the I2 test was below 75% (substantial heterogeneity), the results were pooled. A fixed-effects model was used to analyze and $p<0.05$ was set as a statistical significance. Studies with a risk of bias judged as "high" were excluded from the analysis. A subgroup analysis of treatment-resistant patients (TRD) was also planned.

Bibliogrpahy

1. Higgins, J.; Green, S. Cochrane Handbook for Systematic Reviews of Interventions: Online Version 5.1.0. *Cochrane Colab.* **2011**.
2. Sterne, J. A. C.; Savović, J.; Page, M. J.; Elbers, R. G.; Blencowe, N. S.; Boutron, I.; Cates, C. J.; Cheng, H.-Y.; Corbett, M. S.; Eldridge, S. M.; Emberson, J. R.; Hernán, M. A.; Hopewell, S.; Hróbjartsson, A.; Junqueira, D. R.; Jüni, P.; Kirkham, J. J.; Lasserson, T.; Li, T.; McAleenan, A.; Reeves, B. C.; Shepperd, S.; Shrier, I.; Stewart, L. A.; Tilling, K.; White, I. R.; Whiting, P. F.; Higgins, J. P. T. RoB 2: A Revised Tool for Assessing Risk of Bias in Randomised Trials. *BMJ* **2019**, *366*, l4898. https://doi.org/10.1136/bmj.l4898.
3. Sterne, J.; Higgins, J. Sterne JAC, Higgins JPT, Reeves BC on Behalf of the Development Group for ACROBAT-NRSI. A Cochrane Risk Of Bias Assessment Tool: For Non-Randomized Studies of Interventions (ACROBAT-NRSI), Version 1.0.0, 24 September 2014. Available from Http://Www.Bristo.
4. McGuinness, L. A.; Higgins, J. P. T. Risk-of-Bias VISualization (Robvis): An R Package and Shiny Web App for Visualizing Risk-of-Bias Assessments. *Res. Synth. Methods* **2020**, *n/a* (n/a). https://doi.org/10.1002/jrsm.1411.
