# Supplementary material for: Celecoxib for Mood Disorders: A Systematic Review and Meta-Analysis of Randomized Controlled Trials
Source: J Clin Med. 2023 May 16;12(10):3497. doi: 10.3390/jcm12103497 (PMC10218898; doi:10.3390/jcm12103497)
Supplement: Supplementary file 1 [file jcm-12-03497-s001.zip › Supplementary Material File S2.pdf]

Supplementary Material S2. In vivo studies in animal models of depression and mania (n=19).

Author, year, country	Species, sample size (N), sex (M/F), depression/manic model	Drug tested	Procedure	Result: (1) behavioral test; (2) adverse effects; (3) inflammatory markers
Myint et al., 2007, Netherlands [50]	Sprague Dawley rats, N=8/group, M, OBX rats	CEL 10 mg/kg/day p.o. 2 weeks	OFT, ET<br>IL-1β, TNF-α, IL-10, TGF-β_1, corticosterone	1) CEL reversed hyperactivity in OBX-rats in OFT and ET<br>3) CEL reversed inflammatory activity in brain: OBX + CEL group was lower IL-1β and IL-10 in hypothalamus, lower TNF-α and IL-1β and higher IL-10 in prefrontal cortex compared to OBX group; cytokines and corticosterone were not changed in blood
Guo et al., 2009, China [51]	Sprague Dawley rats, N=10/group, M, CUS	CEL 16/8/2 mg/kg/day p.o. 3 weeks<br>CEL 16 mg/kg p.o. single dose	SPT, OFT<br>PGE2	1) Chronic CEL reversed reduced of sucrose solution intake and sucrose preference in SPT and attenuated anxious behavior in OFT<br>3) Reduced PGE2 level in brain was observed in chronic CEL treatment and single dose
Song et al., 2009, Canada [65]	Sprague Dawley rats, N=10/group, M, OBX rats	CEL 5 mg/kg/day i.p. 4 weeks	OFT<br>IL-1β, PGE2, corticosterone, mRNA NGF expression, PGE2	1) CEL significantly attenuated depressive-like behavior (OFT) in OBX rats<br>3) CEL reduced IL-1β and PGE2 concentration in the serum OBX rats and attenuated PGE2 concentration in hypothalamus; CEL treatment significantly blocked the elevation of serum corticosterone levels in OBX rats; CEL attenuated reduction of NGF expression in hippocampus OBX rats
Prakash et al., 2013, India [52]	Wistar rats, N=6/group, M, LPS treated rats	CEL 10/50 mg/kg/day p.o. 1 week	FST, OFT, LA	1) CEL pretreatment attenuated LPS-induced behavioral changes in OFT, FST, LA
Maciel et al., 2013, Brazil [53]	Swiss mice, N=4-8/group, M, CFA model	CEL 3/15/30 mg/kg p.o. 1 week	TST, FST<br>IL-1β, BDNF	1) CEL (15 and 30mg) decreased depressive-like behavior in TST; CEL + bupropion (both 3 mg) decreased depressive-like behaviors in FST, TST<br>3) CEL (30mg) and CEL + bupropion (each 3mg) reversed increase of IL-1β in whole brain; no significant change in hippocampus and cortex; CEL not interfered with BDNF levels
Kurhe et al., 2014, India [54]	Swiss mice, N=6/group, M, HFD	CEL 15 mg/kg/day p.o. 4 weeks	SPT, FST, TST, EPM, LA	1) CEL reversed depressive-like behaviors in SPT, FST, TST, EPM; LA was not affected in HFD depression model

Santiago et al., 2014, Brazil [55]	Wistar rats, N=8-15/group, M, CMS	CEL 10 mg/kg/day p.o. 3 weeks	SPT	1) CEL reversed depressive-like behaviors in SPT in CMS model of depression
Costa-Nunes et al., 2015, Portugal [56]	C57BL/6N mice, N=10/group, M, Stress-induced anhedonia	CEL 7/15/30/50 mg/kg/day p.o. 4 weeks	TST, FST	1) CEL reversed depressive-like behaviors in TST. CEL did not affect behavioral changes in FST
Fischer et al., 2015, Denmark [57]	Sprague Dawley rats, N=10/group, M, IFNα -induced depression model	CEL 16 mg/kg/day p.o. 1 week	FST, OFT<br>Tryptophan-kynurenine pathway metabolites	1) CEL showed a trend towards decreasing the INFα-induced depression-like behavior in FST. IFNα did not change locomotor activity in OFT<br>3) There was a trend for a down-regulation of kynurenic acid in all treatment groups in hypothalamus, although this did not reach statistical significance
Morgese et al., 2018, Italy [58]	Wistar rats, N=8/group, M, Amyloid β-induced model of depression	CEL 15 mg/kg/day s.c. 8 days	FST	1) CEL prevented amyloid β-induced depressive behaviors in FST
Alboni et al., 2018, Italy [66]	Sprague Dowley rats, N=5-7/group, M, CED	CEL 5 mg/kg/day i.g. 1 week	Escape test	1) CEL + fluoxetine (5 mg/kg/day i.p.) partially reversed stressed-induced escape deficit
Song et al., 2019, China [59]	Wistar rats, N=6-18/group, M, CMS, LPS-induced model	CEL 20 mg/kg/day i.p 5 weeks	SPT, FST<br>PGE2, IL-1β, TNFα, IFNγ, glial activation	1) CEL reduced depressive-like behaviors in SPT, FST in two depression models<br>3) CEL decreased PGE2 in two depression models; CEL prevented glial activation and reversed mRNA expression levels of IL-1β, TNFα, IFNγ in hippocampal DG regions
de Munter et al., 2020, Netherlands [61]	FUS[1-359]-tg mouse, N=7-14/group, M	CEL 30 mg/kg/day p.o. 3 weeks	TST,<br>IL-1β, microgliosis	1) CEL reduced despair in FUS[1-359]-tg mouses<br>3) CEL normalized IL-1β level and microgliosis in the brain FUS[1-359]-tg mouses
Mesripour et al., 2020, Iran [60]	Albino mice, N=6/group, M, IFNα induced depression model	CEL 25, 50 mg/kg/day i.p. 6 days	LA, FST, SPT,	1) Acute CEL (single dose 50 mg/kg) significantly blunted IFNα-induced depression-like behavior in FST, SPT. Sub-acute CEL (six days 25 mg/kg) blunted IFNα-induced depression-like behavior in FST
Feng et al., 2020 [62]	C57BL/6 mice, N=3-8/group, M, Chronic mild stress	Celecoxib 10 mg/kg/day i.p. 4 weeks	SPT, FST<br>glial activation	1) CEL ameliorated depressive-like behaviors in SPT, FST<br>3) CEL normalized glial activation in dentate gyrus (DG) in CMS and attenuated glial activation in LPS-induced model

Mesripour et al., 2021, Iran [63]	NMRI mice, N=6/group, M, CYA-induced model	Celecoxib 25/50 mg/kg/day i.p. 3 days	LA, FST, SPT	1) The co-administration CEL with CYA reduced depression-like behaviors in FST and SPT; not changed LA
Strelakova et al., 2022, Multicenter [64]	C57BL/6N mice, N=13-16/group, M, Chronic mild stress	Celecoxib 30 mg/kg/day i.p. 5 weeks	SPT, FST, FCP	1) CEL prevented development of depressive-like behavior (SPT, FST, FCP) in CMS model
Valvassori et al., 2019, Brazil [68]	Wistar rats, N=12/group, M, dextro-amphetamine (d-AMPH)-induced model of mania	Celecoxib 20 mg/kg/day p.o 1 week	OFT	1) Co-administration of celecoxib with lithium (24 mg/kg/day i.p.) abrogated the effect of d-AMPH; this effect was not observed separately
Valvassori et al., 2019, Brazil [67]	Wistar rats, N=10/group, M, d-AMPH-induced model of mania	Celecoxib 20 mg/kg/day p.o. 1 week	OFT, Il-1β, Il-4, Il-10, TNF-alfa	1) Co-administration Li + CEL abrogated the effect of d-AMPH in OFT; this effect was not observed separately<br>3) Co-administration CEL and lithium (24 mg/kg/day) reversed increased Il-4, Il-10, TNF-alfa in the serum, frontal cortex, striatum; Treatment CEL per se decreased only Il-10 level in the serum of animal, Il-4 in frontal cortex, TNF-alfa in striatum

OFT – open field test, ET – emergency test, FST – forced swimming test, LA – locomotor activity, TST – tail suspension test, CUS – chronic unpredictable stress, SPT – sucrose preference test, EPM – elevated plus maze, FCP – fear conditioning paradigm, CMS – chronic mild stress, CED – chronic escape deficit, OBX – olfactory bulbectomized, CFA – Complete Freund Adjuvant, HFD – high fat diet, FUS – fused in sarcoma protein, TNFα – tumor necrosis factor α, TGF-β – transforming growth factor β, PGE2 –prostaglandin E2, NGF – nerve growth factor, LPS -lipopolysaccharide, BDNF – brain-derived neurotrophic factor, IFNα – interferon α, IFNγ – interferon γ, DG – dentate gyrus, CYA – Cyclosporine A, d-AMPH – dextroamphetamine, Li – lithium, IL – interleukin, CEL – celecoxib, CYA – cyclosporine A, FCP – fear conditioning paradigm s.c. – subcutaneously, p.o. – per os, i.p. – intraperitoneal, i.g. – intragastric
